# A gene expression network analysis of the pancreatic islets from lean and obese mice identifies complement 1q like-3 secreted protein as a regulator of β-cell function

**DOI:** 10.1038/s41598-019-46219-3

**Published:** 2019-07-12

**Authors:** James E. Koltes, Itika Arora, Rajesh Gupta, Dan C. Nguyen, Michael Schaid, Jeong-a Kim, Michelle E. Kimple, Sushant Bhatnagar

**Affiliations:** 1 Department of Animal Science, Iowa State University, Ames, IA 50011 USA; 2 Division of Endocrinology, Diabetes, and Metabolism, Department of Medicine and Comprehensive Diabetes Center, University of Alabama, Birmingham, AL 35294 USA; 3 Interdisciplinary Graduate Program in Nutritional Sciences, University of Wisconsin-Madison College of Agriculture and Life Sciences, Madison, WI 53706 USA; 4 Divison of Endocrinology, Diabetes, and Metabolism, Department of Medicine, University of Wisconsin-Madison School of Medicine and Public Health, Madison, WI 53705 USA; 5 Department of Cell and Regenerative Biology, University of Wisconsin-Madison School of Medicine and Public Health, Madison, WI 53705 USA; 6 Department of Academic Affairs, University of Wisconsin-Madison School of Medicine and Public Health, Madison, WI 53705 USA; 7 Research Service, William S Middleton Memorial VA Hospital, Madison, WI 53705 USA

**Keywords:** Cellular signalling networks, Functional clustering, Gene regulatory networks

## Abstract

Secreted proteins are important metabolic regulators. Identifying and characterizing the role of secreted proteins from small tissue depots such as islets of Langerhans, which are required for the proper control of whole-body energy metabolism, remains challenging. Our objective was to identify islet-derived secreted proteins that affect islet function in obesity. Lean and obese mouse islet expression data were analyzed by weighted gene co-expression network analysis (WGCNA) to identify trait-associated modules. Subsequently, genes within these modules were filtered for transcripts that encode for secreted proteins based on intramodular connectivity, module membership, and differential expression. Complement 1q like-3 (C1ql3) secreted protein was identified as a hub gene affecting islet function in obesity. Co-expression network, hierarchal clustering, and gene-ontology based approaches identified a putative role for C1ql3 in regulating β-cell insulin secretion. Biological validation shows that C1ql3 is expressed in β-cells, it inhibits insulin secretion and key genes that are involved in β-cell function. Moreover, the increased expression of C1ql3 is correlated with the reduced insulin secretion in islets of obese mice. Herein, we demonstrate a streamlined approach to effectively screen and determine the function of secreted proteins in islets, and identified C1ql3 as a putative contributor to reduced insulin secretion in obesity, linking C1ql3 to an increased susceptibility to type 2 diabetes.

## Introduction

Secreted proteins reflect the dynamic changes that occur in tissues and serve as within- and across-tissue metabolic regulators^1^. The expression of secreted proteins can be affected by a wide array of stimuli, including development, maturation, environmental, physiological, and pathological states. Upon secretion, these proteins relay the metabolic status of their tissue of origin *via* endocrine, paracrine, or autocrine signaling to impart a regulatory effect on their target tissue.

Alterations in secreted protein-regulated signaling pathways (e.g., leptin, adiponectin, IL-6) have been associated with increased susceptibility to obesity-associated metabolic disorders such as type 2 diabetes (T2D)^2–5^. The functional characterization of these proteins has improved our understanding of the mechanisms underlying obesity and its co-morbidities. However, the role of most annotated secreted proteins as metabolic regulators remains unknown.

Several techniques such as antibody bead-based arrays^6^, mass spectrometry^7^, secretion traps^8^, aptamer-based methods^9^, and computational algorithms^10^ have been used to identify secreted proteins. Even with recent advancements in mass spectrometry, the identification of secreted proteins in serum or tissue samples has remained challenging, due, in part, to their relatively low protein abundance^11^. This limitation has been attributed to their short half-life, a naturally restricted contribution from tissues that are relatively small in mass, and the absence of stimuli required for their secretion during experimental sampling. Furthermore, even after a secreted protein is identified, the difficulty remains in identifying cellular pathways affected in the target tissue for downstream functional analyses. An experimental paradigm allowing for the analysis of secreted protein’s ‘tissue of origin’ and downstream functional profiling of affected pathways in the target tissues would be a great advance in the identification and characterization of candidate secreted proteins.

The islets of Langerhans, which are required for the proper control of whole-body energy metabolism, are mini-organs comprising of ~1% of the total pancreas weight. The α-, β-, and δ-cells of the pancreatic islets secrete key metabolic hormones such as glucagon, insulin, and somatostatin, respectively, to maintain whole-body glucose homeostasis in the fed and fasted states^12^. Increased metabolic stress due to obesity and its commonly-associated inflammation and peripheral insulin resistance causes islet cells to functionally adapt by altering their hormone secretion to maintain blood glucose levels^13–15^. Failure of an initial adaptation or continued metabolic stress disrupts the islet micro-environment and impairs the coordinated secretion of islet hormones, affecting whole-body energy metabolism. Yet, in some obese individuals, a robust β-cell adaptation response prevents the progression to T2D^16–18^. Thus, identifying signaling pathways within islets that control the adaptation of the islet cells in response to obesity will provide insights into the mechanisms underlying T2D.

C57BL/6J mice homozygous for the *Leptin*^*Ob*^ mutation (B6-Ob) exhibit hyperphagia, leading to morbid obesity and severe insulin resistance. B6-Ob mice progress from β-cell compensation to β-cell failure, acting as strong models of the natural course of human T2D. Phenotypic comparisons between B6-Ob mice and their lean controls (B6-lean) have been incredibly useful in identifying novel cellular pathways and heritable traits affecting energy metabolism^19^.

Co-expression network-based approaches enable a systematic investigation of functional protein changes in a complex disease like obesity^20^. Highly correlated genes that are either co-activated or co-repressed in different disease states form a gene network. In this work, we employed weighted gene co-expression network analysis (WGCNA) and gene set enrichment analysis (GSEA) to identify secreted proteins from islets of B6-lean and B6-Ob mice that regulate islet function in response to obesity and insulin resistance. Our objective for this study was to screen for islet-derived secreted proteins that would differentially regulate islet function in healthy and disease states in an autocrine or paracrine manner. We hypothesized that specific islet-derived secreted proteins are critical for the proper function of the islet micro-environment, which become dysfunctional in the obese, pre-diabetic state.

## Results

### Generation of gene modules in islets

**Figure 1:**
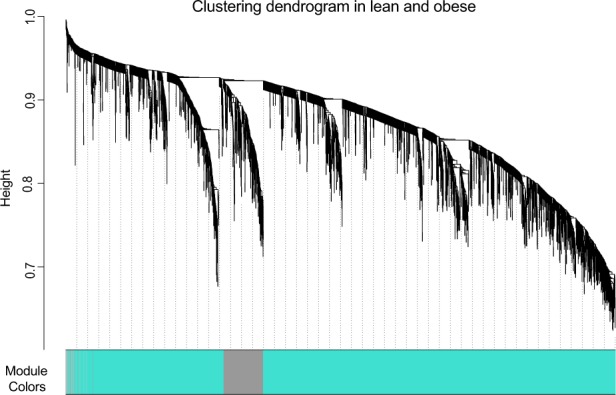
Gene dendrogram showing co-expression modules in the pancreatic islets of lean and obese mice. Hierarchical clustering was performed for the merged lean and obese islet expression data (*N*_*samples*_ = 40 *and N*_*Genes*_ = 40639). The branches in the dendrogram denote a cluster of genes. Clusters of highly co-expressed genes are represented as color-coded modules (*N*_*modules*_ = 28) shown along the horizontal x-axis. The turquoise module was identified as the major gene cluster (*N*_*Genes*_ = 11682), and the grey module represents unassigned genes. The vertical y-axis shows the co-expression distance between genes identified in the dendrogram.

**Figure 2:**
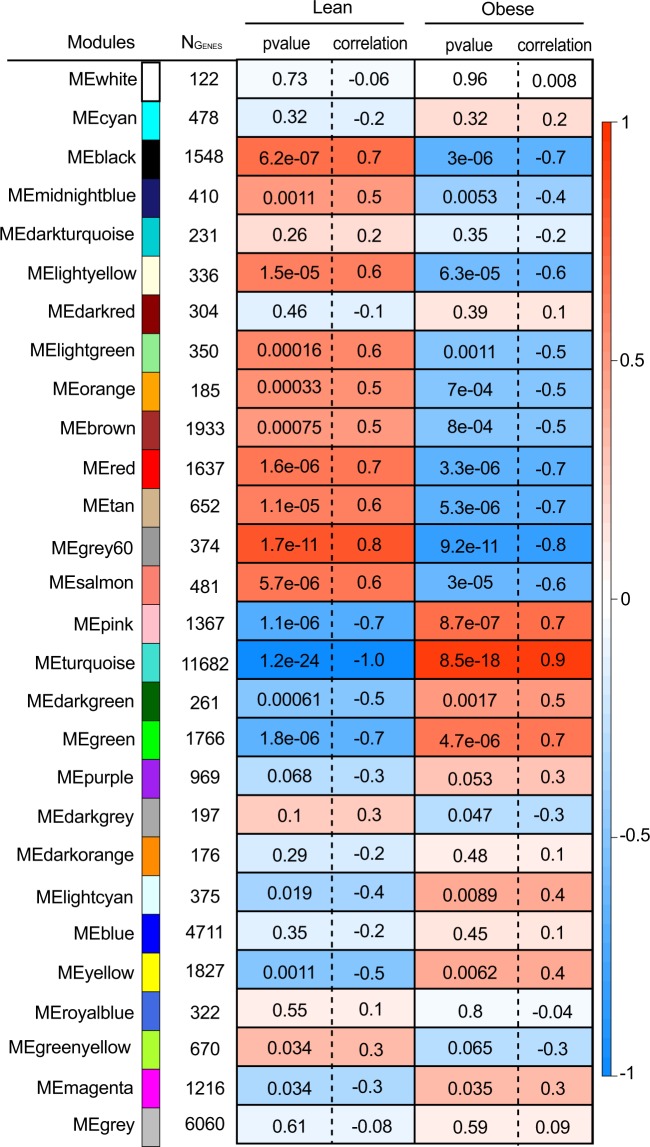
Module-trait relationships in the islets of lean and obese mice. Each row corresponds to the 28 different color-coded modules identified by WGCNA. The number of genes forming each module are shown in “N_Genes_.” Cells for the lean and obese phenotype contain the corresponding p-value and correlation coefficient for the module. The color of each cell is assigned based on the correlation value of the module; dark red represents a strong positive correlation and dark blue represents a strong negative correlation.

We obtained gene expression data from islets of 20 B6-lean and 20 B6-Ob mice^21^. Hierarchical clustering identified no outliers (Supplementary Fig. S1); therefore, all samples were included in this study. To identify gene expression modules, WGCNA based on Pearson’s correlation was performed, identifying a soft threshold of β = 6^20,22,23^. This cut-off was used in subsequent analysis to generate gene expression modules (Supplementary Fig. S2). A total of 28 modules were identified (Fig. 1 and Table 1). Of these, 18 modules were identified to be significantly associated with lean and obese states (p < 0.05). A heat map for the module-trait relationship is shown in Figure 2.

**Table 1:** Modules identified by analyzing merged lean and obese islet expression data by WGCNA.

Modules	Size (No. of genes)	Lean		Obese	
		P-value	Correlation	P-value	Correlation
Turquoise	11682	1.18E-24	−0.97	8.49E-18	0.93
Grey60	374	1.67E-11	0.84	9.22E-11	−0.82
Black	1548	6.19E-07	0.70	3.05E-06	−0.66
Pink	1367	1.08E-06	−0.69	8.67E-07	0.69
Red	1637	1.60E-06	0.68	3.32E-06	−0.66
Green	1766	1.84E-06	−0.67	4.67E-06	0.65
Salmon	481	5.71E-06	0.65	2.97E-05	−0.61
Tan	652	1.14E-05	0.63	5.32E-06	−0.65
Light yellow	336	1.53E-05	0.63	6.35E-05	−0.59
Light green	350	1.62E-04	0.56	1.12E-03	−0.50
Orange	185	3.33E-04	0.54	6.97E-04	−0.51
Dark green	261	6.14E-04	−0.52	1.67E-03	0.48
Brown	1933	7.47E-04	0.51	7.96E-04	−0.51
Yellow	1827	1.09E-03	−0.50	6.16E-03	0.43
Midnight blue	410	1.10E-03	0.50	5.27E-03	−0.43
Light cyan	375	1.85E-02	−0.37	8.94E-03	0.41
Green yellow	670	3.37E-02	0.34	6.45E-02	−0.30
Magenta	1216	3.40E-02	−0.34	3.45E-02	0.34
Purple	969	6.75E-02	−0.29	5.29E-02	0.31
Dark grey	197	1.01E-01	0.26	4.72E-02	−0.32
Dark turquoise	231	2.59E-01	0.18	3.55E-01	−0.15
Dark orange	176	2.91E-01	−0.17	4.77E-01	0.12
Cyan	478	3.25E-01	−0.16	3.22E-01	0.16
Blue	4711	3.48E-01	−0.15	4.47E-01	0.12
Dark red	304	4.58E-01	−0.12	3.94E-01	0.14
Royal blue	322	5.51E-01	0.10	8.03E-01	−0.04
Grey	6060	6.07E-01	−0.08	5.90E-01	0.09
White	122	7.25E-01	−0.06	9.62E-01	0.01

### Identification of intramodular connectivity (IMC) and module membership (MM) for genes within the enriched modules

The IMC was calculated to determine how well a gene is connected with the other genes in the enriched modules (k_Total_). We also calculated module membership (MM) to determine how well the expression of each gene is correlated with the ‘module-eigengene value,’ calculated by performing a principal component analysis for each module. A total of 15823 unique genes were identified that enriched in the lean and obese modules. Of these, 3956 genes had high k_Total_ (Supplementary Table S1). Moreover, 3292 genes had a MM value > 0.8 and were thus categorized as “hub genes” (Supplementary Table S1). Through this analysis, a total of 1835 genes were identified with high IMC and MM in islets from B6-lean *vs*. B6-Ob mice (Supplementary Table S1). This list served as a reference list for the identification of the candidate secreted proteins.

### Identification of candidate secreted protein regulators

**Figure 3:**
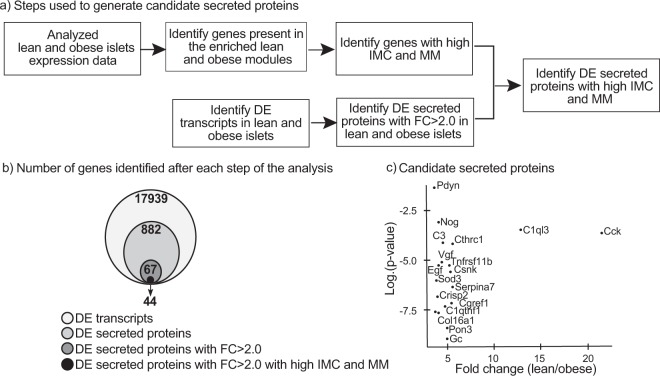
Filtering and identification of candidate islet secreted proteins. (**a**) Experimental paradigm for identifying genes for annotated secreted proteins DE in islets with obesity, high MM (>0.8), and high IMC (Z > 1.96). (**b**) Venn diagram showing the number of transcripts identified after each step of the analysis. The circles are proportional to the number of transcripts remaining after each filter is applied. (**c**) Graph representing top (N = 18) candidate secreted proteins that are DE in islets from obese *vs.* lean mice. The x-axis represents the fold change in the mRNA abundance with obesity, while the y-axis represents the log (p-value) change in mRNA expression.

B6-lean and B6-Ob islet gene expression data were filtered for secreted proteins based on the differential expression (DE), MM, and IMC (Fig. 3a). A total of 2351 transcripts annotated as secreted proteins in the UniProt database were used in the analysis (Supplementary Table S2). This list was curated based on the presence of a secretory signal sequence in the protein. Using B6-lean and B6-Ob islet expression data, a total of 17939 transcripts were identified to be DE with obesity (false discovery rate, FDR < 5%; Supplementary Table S3). Of these, 882 secreted protein transcripts were identified as DE (q < 0.05), and among them, 67 genes had a fold change (FC) > 2.0 in islets (Supplementary Table S4). These transcripts were subsequently filtered for hub genes (MM > 0.8) with high IMC by using the reference list that was identified from the lean and obese enriched modules (Supplementary Table S1). Through these steps, 44 transcripts were identified that encode for secreted proteins that were DE, had high MM with high IMC. This generated a list of candidate regulatory secreted proteins (Supplementary Table S5). A Venn diagram shows the number of genes that were filtered at each step based on the analysis (Fig. 3b). Cholecystokinin (Cck) and complement 1q-like 3 (C1ql3) were identified to be among the top DE transcripts, with FC of 21.69 and 12.90, respectively (Fig. 3c). The top 20 secreted protein regulators identified by using this filtering approach are shown in Table 2. As the role of Cck in islet function has been well characterized^24–27^, we focused on C1ql3 for downstream analysis.

**Table 2:** The top 20 secreted protein regulators identified by analyzing merged islet expression data from lean and obese mice.

Gene Symbol	Module member ship (MM)	Intra modular connectivity (k_Total_)	Q-value for the differential expression (Obese *vs*. Lean)	Gene expression fold change (Obese *vs*. Lean)
Cck	0.86	3806.50	2.52E-04	21.69
C1ql3	0.87	3823.40	3.72E-04	12.90
BC027127	0.89	2667.72	1.93E-07	9.55
Cthrc1	0.94	4343.40	6.85E-05	5.57
Serpina7	0.90	4089.56	3.77E-07	5.49
Cgref1	0.99	4793.24	7.62E-08	5.40
Csnk	0.96	4460.55	2.79E-06	5.38
Gc	0.96	4462.27	1.00E-09	4.95
Pon3	0.96	4466.18	3.00E-09	4.95
C3	0.90	3863.55	7.04E-05	4.43
Vgf	0.95	4528.39	7.38E-06	4.34
Crisp2	0.93	4317.46	1.42E-07	3.94
Sod3	0.93	4109.99	9.62E-07	3.80
Prss23	0.82	3295.50	2.42E-08	3.69
Pdyn	0.81	3442.96	4.72E-02	3.66
Insl6	0.88	4139.31	9.73E-07	3.59
Sema3c	0.96	4513.74	5.43E-07	3.57
Creld2	0.88	4044.29	7.96E-06	3.57
Ptprn	0.92	4266.80	6.05E-06	3.41
Pycr1	0.95	4638.24	7.41E-08	3.35

The candidate regulators were ranked based on the fold-change in the mRNA expression. The corresponding q-value for DE, MM, and IMC (k_Total_) values are shown in the table.

### Identification of candidate secreted proteins by separately analyzing lean and obese islet expression data

**Figure 4:**
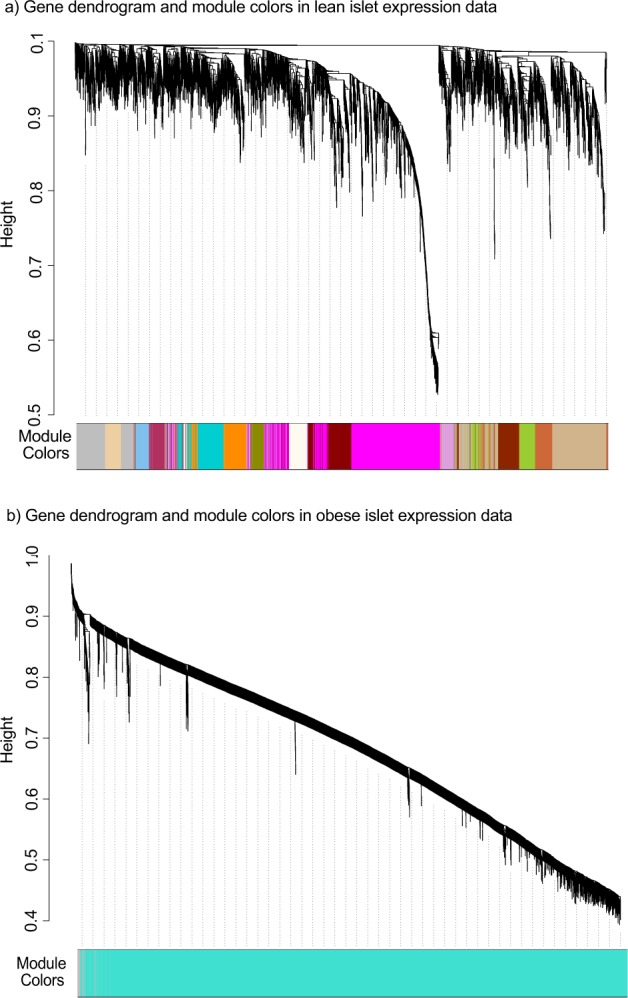
Gene dendrogram showing gene co-expression modules in islets from lean (**a**) and obese (**b**) mice. The branches of the dendrogram denote clusters of genes. Clusters of highly co-expressed genes are represented as color-coded modules shown along the horizontal x-axis. The vertical y-axis indicates the co-expression distance between the genes identified in the dendrogram. The grey module represents unassigned genes.

**Figure 5:**
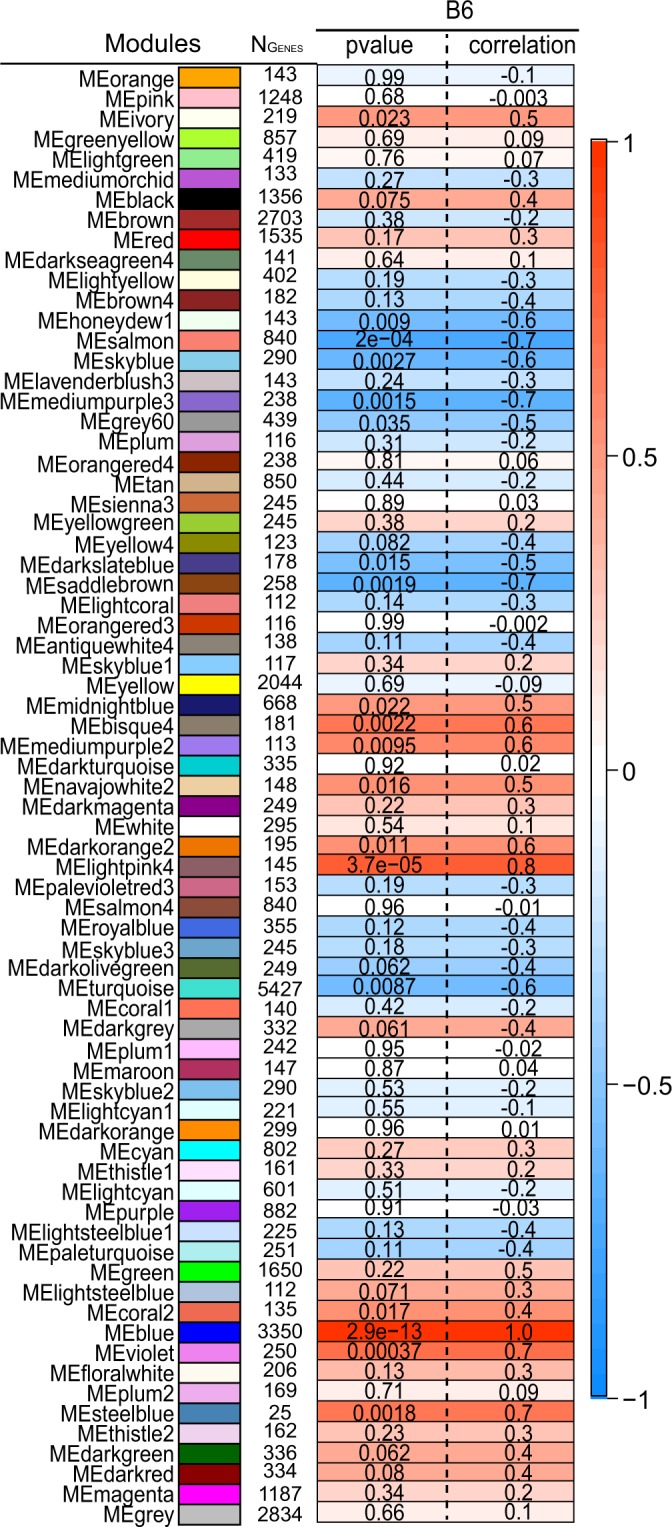
Module-trait relationships in islets of lean mice. Each row corresponds to the 72 different color-coded modules identified by WGCNA. N_Genes_ is the number of genes in each module. Neighboring cells show the corresponding p-value and correlation coefficient for the module. Dark red represents a strong positive correlation and dark blue represents a strong negative correlation.

**Figure 6:**
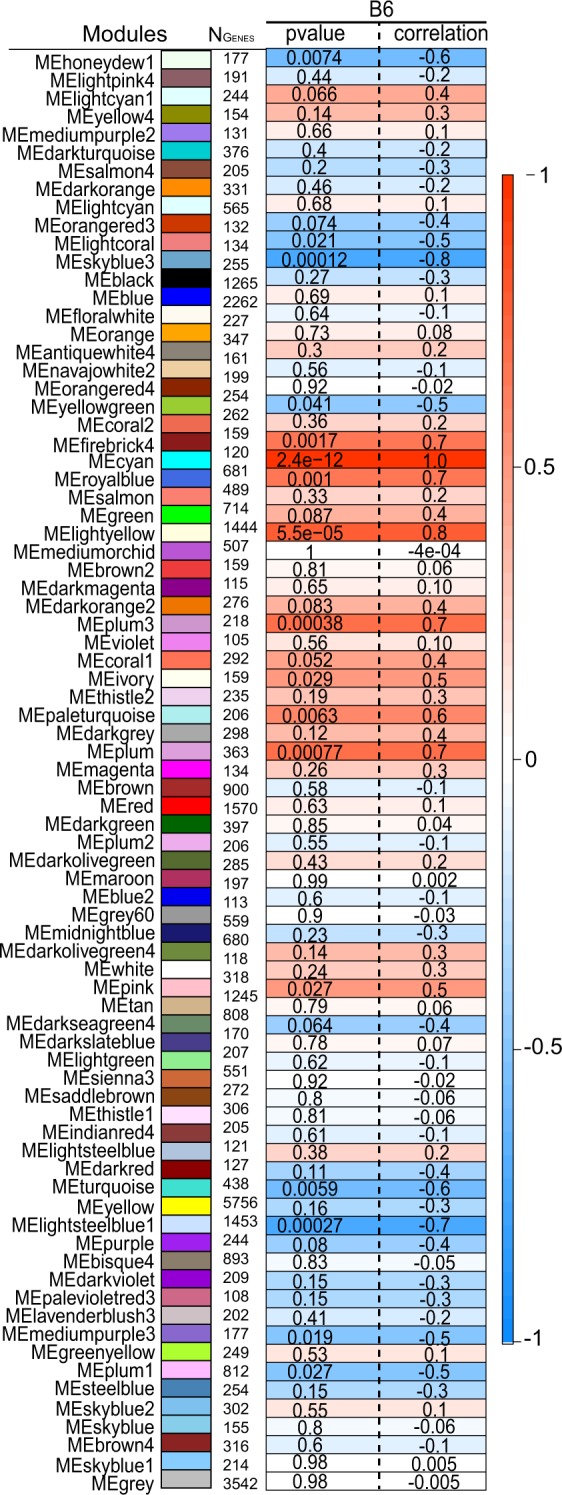
Module-trait relationships in islets of obese mice. Each row corresponds to the 78 different color-coded modules identified by WGCNA. N_Genes_ is the number of genes in each module. Neighboring cells show the corresponding p-value and correlation coefficient for the module. The color of each cell is assigned based on the correlation value of the module; dark red represents strong positive correlation and dark blue represents a strong negative correlation.

**Figure 7:**
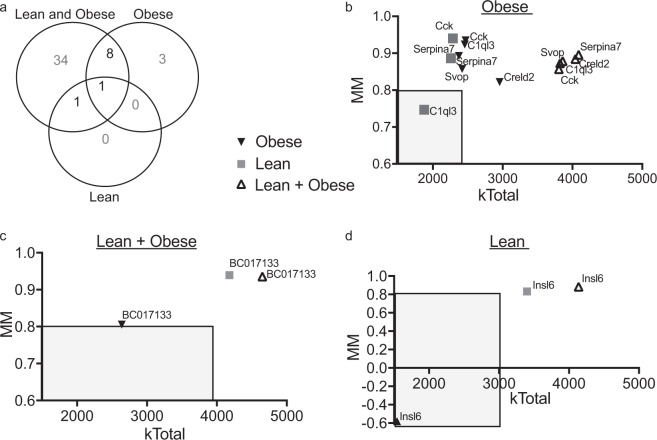
Filtering and identification of candidate secreted protein regulators of islet function in obesity. (**a**) Venn diagram showing the overlap between secreted protein regulators identified by analyzing islet expression data from lean, obese, and merged (lean plus obese) data. The numbers in **bold** indicate candidate regulators for islet function in lean, obese, and either state. The plot of MM *vs.* IMC (k_Total_) values for secreted protein regulators in islets of obese, both lean and obese, and lean mice are shown in *b*, *c*, and *d*, respectively. The shaded area in the graph shows the threshold cutoff values.

To determine whether C1ql3 differentially affects islet function in lean *vs*. obese mice, gene co-expression modules were generated separately for lean and obese mouse islet expression data. A total of 72 modules were identified in islets from lean mice and 78 modules in islets from obese mice (Fig. 4b). Of these modules, 19 in lean (Fig. 5 and Supplementary Table S6) and 17 in obese (Fig. 6 and Supplementary Table S7) were significantly associated with clinical traits (p < 0.05). These modules corresponded with 6394 and 10231 genes in the lean- *vs*. obese-enriched modules, respectively (Supplementary Table S8–S9). Of these, 2021 genes in lean and 1515 genes in obese had high IMC (k_Total_) with MM greater than 0.80 (Supplementary Tables S8 and S9, respectively). These lists were further filtered for the 67 secreted protein transcripts that were identified to be DE with FC > 2.0 (Supplementary Table S4). Based on this analysis, 2 secreted protein transcripts, *Insl6 and BC017133*, were identified as hub genes in islets of lean mice (Supplementary Table S10) and 12 genes as hub genes in islets of obese mice (Table 3 and Supplementary Table S11) (Fig. 7a).

**Table 3:** Secreted protein regulators identified by analyzing islet gene expression data from obese mice.

Gene Symbol	Module member ship (MM)	Intra modular connectivity (k_Total_)	Q-value for the differential expression (Obese *vs*. Lean)	Gene expression fold change (Obese *vs*. Lean)
Cck	0.93	2469.23	2.52E-04	21.69
C1ql3	0.92	2458.39	3.72E-04	12.90
Serpina7	0.89	2372.95	3.77E-07	5.49
Creld2	0.82	2960.94	7.96E-06	3.57
C630007B19Rik	0.82	2799.11	3.47E-06	2.81
Svop	0.86	2423.68	2.34E-04	2.45
Smoc1	0.93	2647.30	1.40E-05	2.43
Tgfb3	0.93	2390.97	1.20E-09	2.39
BC017133	0.81	2643.02	4.88E-07	2.27
Dcx	0.89	3053.80	1.39E-05	2.23
Serpini1	0.94	2384.99	2.71E-07	2.19
Kazald1	0.96	2535.36	4.34E-05	2.17

The candidate regulators were ranked based on the fold-change in the mRNA expression with obesity. The corresponding q-value for DE, MM, and IMC (k_Total_) values are shown in the table.

Next, we determined secreted protein regulators that might differentially affect islet function in the lean *vs*. obese state. For this, candidate secreted protein regulators that were identified individually to affect islet function in lean and obese mice (Table 3 and Supplementary Table S11) were compared to 44 secreted protein regulators, which were identified after analyzing merged lean and obese islet gene expression data (Fig. 7a, Supplementary Table S5). Using a Venn diagram, *Insl6* was identified as a regulator of islet function in lean (Fig. 7d), while *Cck, C1ql3, Serpina7, Creld2, Svop, Smoc1, Tgfβ3, and Serpini1* were identified as regulators of islet function in obese (Fig. 7b). Moreover, *BC017133* was identified as a regulator of islet function in both lean and obese mice (Fig. 7c). The ranking of these putative regulators based on FC is shown in Table 4. *C1ql3* was identified as one of the top regulators of islet function specifically in obesity, with an IMC of 2458 and MM of 0.92. Both these attributes were significantly above their respective threshold values for the analysis. Moreover, when the lean and obese samples were analyzed together, the IMC and MM values were 3823.40 and 0.86, respectively, for C1ql3. These results suggest that C1ql3 has high connectivity and likely functions as a hub gene in the regulation of islet function in the obese state.

**Table 4:** Identified candidate secreted protein regulators to affect islet function in lean, obese, and those common to lean and obese.

Gene Symbol	Gene Name	Module member ship (MM)	Intra modular connectivity (k_Total_)	Q-value for differential expression (Obese *vs*. Lean)	Gene expression fold change (Obese *vs*. Lean)
**Secreted protein regulators of islet function in obesity**					
Cck	Cholecystokinin	0.93	2469.23	2.52E-04	21.69
C1ql3	Complement 1q like-3	0.92	2458.39	3.72E-04	12.90
Serpina7	Serine (or cysteine) peptidase inhibitor, clade A member 7	0.89	2372.95	3.77E-07	5.49
Creld2	Cysteine-rich with EGF-like domains 2	0.82	2960.94	7.96E-06	3.57
Svop	SV2 related protein	0.86	2423.68	2.34E-04	2.45
Smoc1	SPARC related modular calcium binding 1	0.93	2647.30	1.40E-05	2.43
Tgfb3	Transforming growth factor, beta 3	0.93	2390.97	1.20E-09	2.39
Serpini1	Serine (or cysteine) peptidase inhibitor, clade I, member 1	0.94	2384.99	2.71E-07	2.19
**Secreted protein regulator of islet function in lean**					
Insl6	Insulin-like 6	0.83	3401.01	9.73E-07	3.59
**Secreted protein regulator of islet function in lean and obesity**					
		Value (Lean: Obese)	Value (Lean: Obese)		
BC027127	cD. sequence BC027127	0.91	2359.03	1.93E-07	9.55

The regulators were ranked based on the fold change. The corresponding IMC (k_Total_), MM, and DE values are also shown the in the table.

### Gene set enrichment analysis associates C1ql3 with the islet secretory response

**Figure 8:**
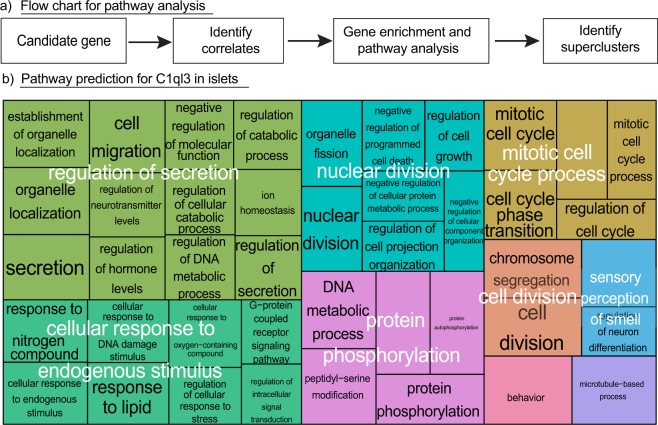
Pathway prediction for *C1ql3* in islets. (**a**) Schematic showing the steps taken to identify cellular pathways in islets for candidate secreted proteins. (**b**) A tree plot generated by REVIGO software showing enriched functions for the islet C1ql3. Each large colored rectangle in a tree plot represents a supercluster composed of grouped GO terms. Superclusters are indicated in white text. The small rectangles of GO terms are indicated in black text. The size of the supercluster is based on the frequency of individual GO terms in the analysis.

Gene set enrichment analysis (GSEA) was performed to predict the cellular function of candidate secreted proteins. The steps used in determining the islet function of C1ql3 are shown in Fig. 8a. Briefly, *C1ql3* within-islet correlations were determined using lean and obese expression data, and transcripts with a correlation value > |0.5| were selected for the gene ontology (GO) enrichment analysis, generating GO terms. The GO terms containing fewer than 200 genes within a GO term (Bonferroni adjusted p < 0.05) were selected for the identification of cellular pathways affected by C1ql3 (Supplementary Tables S12, S13). Selected GO terms were subjected to REVIGO^28^ to generate superclusters, enabling the prediction of C1ql3 function (Supplementary Table S14 and Fig. 8b). Each supercluster is represented by a colored rectangle in a tree plot (Fig. 8b). ‘Regulation of secretion’ was identified as a major islet supercluster for C1ql3, comprised predominately of GO terms associated with secretory processes. Smaller superclusters were also identified pertaining to nuclear division, mitotic cell cycle process, and the cellular response to endogenous stimuli (Fig. 8b). The REVIGO analysis output confirmed *C1ql3* correlates primarily with islet secretory function (Fig. 8). To determine the specificity of this analysis in predicting C1ql3 function, GSEA and REVIGO were performed for islet-derived trefoil factor 2 (*Tff2*) and somatostatin (*Sst*): *Tff2* served as a negative control, as there was no enrichment for the secretory process in islets, while Sst, secreted from δ-cells and a long-known regulator of islet hormone secretion^29^, served as a positive control (Supplementary Fig. S3). Moreover, no significant clusters were identified in islets for *Ins1* (data not shown). These outcomes suggest that the identification of C1ql3 in a ‘regulation of secretion’ supercluster was not due to the intrinsic nature of islets as a secretory tissue (Supplementary Table S15 and Fig. S3a).

### Hierarchical clustering of *C1ql3* correlates enriched for secretory processes

**Figure 9:**
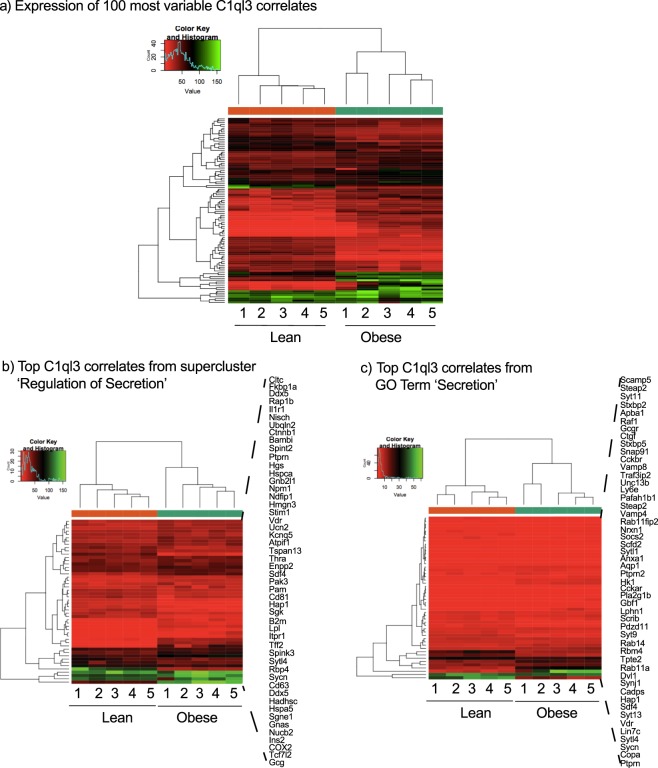
Hierarchical clustering of *C1ql3* correlates in islets from 10-week-old, B6-lean *vs*. B6-Ob mice. (**a**) A heat map of clustered genes showing the most variably expressed genes between lean and obese islets. Expression values are representative of the full dataset and were selected purely based on ability to differentiate lean *vs*. obese islets. (**b**) Heat map of gene expression patterns for all differentially-expressed correlates of *C1ql3* ≥ |0.5| that enriched for secretion-related GO terms as clustered by REVIGO (see Fig. 8). (**c**) Heat map of gene expression patters for all differentially-expressed correlates of *C1ql3* ≥ |0.5| that enriched for the specific term GO term ‘secretion.’

Hierarchical clustering (hclust function in R) was used to identify differences in gene expression between lean and obese mouse islets, showing *C1ql3* correlates (as defined by the genefilter function in R) differentiate lean and obese animals by gene expression heat maps. Interestingly, many of the *C1ql3* correlates that differentiate lean and obese mice were related to the secretory processes (Fig. 9a). As a comparison, Figure 9b-c show that the most variable gene expression values were for the correlates associated with secretory terms that clustered in a tree plot into ‘regulation of secretion’ (enrichment significance: Bonferroni adjusted p < 0.05) and the specific ontology term ‘secretion’ (enrichment significance: Bonferroni adjusted p < 0.05), respectively. We also clustered the most variably expressed genes across the entire islet gene expression dataset (Fig. 9a). The similarity between all graphs may indicate that some of the difference may be related to the difference in the factors affecting insulin secretion.

Interestingly, clusters of *C1ql3*-correlated genes enriched for secretion terms appear to differentiate lean and obese mice. Several genes having disparate roles related to insulin secretion, including *Nucb2, Ins2, Cox2, Tcf7l2*, and *Gcg*, were identified across all secreted ontology terms clustered by REVIGO (Fig. 9b). For the specific ontology term ‘secretion’, the genes included *Ptprn, Sycn, Sytl4, Lin7c, Vdr, Syt13, Sdf4, Rab11a, Syt9, Pdzd11, Cckar, Nrxn1, Vamp4, Unc13b (Munc13), Snap9, Gcgr, Stxbp5, Syt11, Stxbp2* and *Hap1* (Fig. 9c and Supplementary Table S16). Among the genes that were most variably expressed across all *C1ql3* correlates between lean and obese animals were many of these same genes, as well as *Slc2a2, Klk1, Dmbt1, Rnase1, Rbp4, Itm2c, Med31, ATP6, PEG3, Ppu, Cpa1, Reg1, Cpb1, Prss2, Ptchd2*, and *lapp* (Fig. 9a). Many of these genes have previously known roles in regulating metabolic processes related to obesity, diabetes, and specific processes related to insulin secretion. Altogether, these results indicate that in islets, *C1ql3* is directly involved or intimately related to pathways associated with secretion and is correlated with genes involved specifically in insulin secretion.

### Biological validation identified the role of C1ql3 in regulating insulin secretion

**Figure 10:**
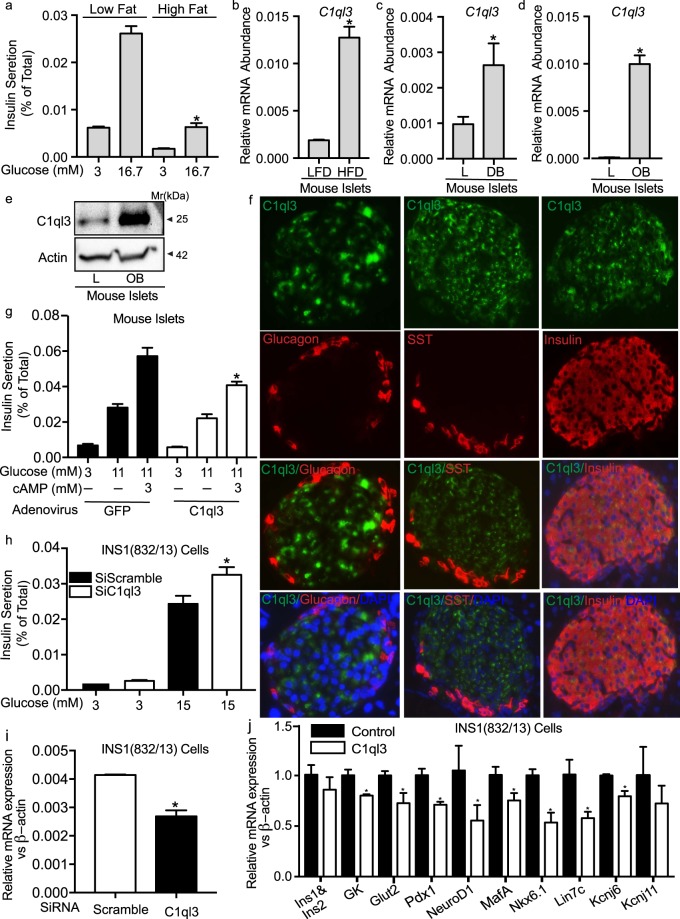
C1ql3 regulates β-cell function. (**a**) Insulin secretion reported as a percent of total (%Total) was performed in islets obtained from lean and high fat diet-induced obese B6 mice. Values are means ± S.E. of N ≥ 3. *p ≤ 0.05 for the glucose-stimulated insulin secretion from islets obtained from obese *vs*. lean mice. The relative mRNA abundance of *C1ql3* using SybrGreen qPCR, as calculated by ∆Ct *vs.* *β-actin* in (**b**) islets from lean and high-fat diet induced obese B6 mice, (**c**) islets from lean and *db/db* obese B6 mice, and (**d**) islets from lean and B6-Ob mice. Values are means ± S.E. of ≥3. *p ≤ 0.05 for the mRNA abundance of *C1ql3* in islets of obese *vs*. lean mice. (**e**) Western blot analysis showing the protein abundance in islets of lean and B6-Ob mice. Representative blot is from N = 3 experiments. (**f**) Immunofluorescence was performed in mouse pancreatic tissue obtained from B6 mice. Anti-insulin, -glucagon, and -somatostatin (SST) antibodies were used to mark β-, α-, and δ-cells, respectively. DAPI (blue) for nuclei staining and anti-C1ql3 antibody (green) was used to assess the expression of C1ql3. (Scale Bar = 10 µm). Representative images are from N = 4 experiments (**g**) Insulin secretion was performed in islets expressing C1ql3 or GFP, which was achieved *via* infection by adenovirus. Values are means ± S.E. of N ≥ 3 experiments. *p ≤ 0.05 for the insulin secretion in response to C1ql3 *vs*. GFP control. (**h**) Insulin secretion was performed in INS1(832/13) β-cells subjected to transfection by siC1ql3 or SiScramble RNA. Values are means ± S.E. of N ≥ 3 experiments. *p ≤ 0.05 for the insulin secretion in response to siC1ql3 *vs*. siScramble control. (**i**) The relative mRNA abundance of *C1ql3* in INS1(832/13) β-cells expressing siC1ql3 *vs*. siScramble using SybrGreen qPCR, as calculated by ∆Ct vs. *β-actin*. Values are means ± S.E. of N ≥ 3 experiments. *p ≤ 0.05 for the *C1ql3* mRNA abundance in response to siC1ql3 *vs*. siScramble control. (**j**) The relative mRNA abundance of genes determined in INS 1(832/13) β-cells transected with either C1ql3 or GFP. Values are means ± S.E. of ≥ 3. *p ≤ 0.05 for the mRNA abundance of genes in response to overexpression of *C1ql3 vs*. GFP in INS1(832/13).

Microarray expression data for *C1ql3* was confirmed by real-time quantitative PCR. An increase in the relative mRNA abundance of *C1ql3* was observed in islets of several mouse models of obesity compared to their lean controls. This included greater than 4-fold increase in islets of high-fat-diet-fed obese B6 mice *vs*. low-fat-diet-fed B6 control mice; ~2.5-fold increase in islets of B6^*db/db*^ *vs*. B6-lean mice, and ~64-fold increase in islets of B6-Ob *vs*. B6-lean mice (Fig. 10b–d). Moreover, a significant increase in C1ql3 protein levels was observed in the islets of B6-Ob *vs*. B6-lean mice (Fig. 10e). The increased expression of C1ql3 in islets is correlated with a reduction in glucose-stimulated insulin secretion at 16.7 mM from islets of high-fat-diet-fed obese B6 mice *vs*. low-fat diet-fed B6 control mice (Fig. 10a).

Islets are comprised primarily of insulin secreting β-cells, glucagon-secreting α-cells, and somatostatin (SST)-secreting δ-cells. Therefore, we determined the islet cell types that express C1ql3 by immunohistochemistry. Our results show that C1ql3 (green) co-stained with insulin (red) secreting β-cells (Fig. 10f, right panel). C1ql3 did not co-express with glucagon (Fig. 10f, left panel) or SST ((Fig. 10f, middle panel) positive cells. We next determined the effect of C1ql3 on insulin secretion. Overexpressing C1ql3 *via* adenoviral expression in mouse islets decreased insulin secretion as compared to GFP expressing control islets in response to cAMP (3 mM) at 11 mM glucose (a submaximal stimulatory concentration of glucose). C1ql3 had no inhibitory effect on insulin secretion at basal (3 mM) or 11 mM glucose concentrations. We investigated the effect of C1ql3 knockdown on glucose-stimulated insulin secretion (GSIS) from the INS1(832/13) β-cell line. A significant reduction in *C1ql3* expression was observed with small interfering (si) RNA targeting of C1ql3 as compared to a scrambled siRNA control (Fig. 10i). This reduction in C1ql3 expression correlated with increased INS1(832/13) insulin secretion in response to 15 mM glucose (Fig. 10h). However, knockdown of C1ql3 had no effect on INS1(832/13) insulin secretion at a basal (3 mM) glucose concentration. These results show that C1ql3 is expressed in β-cells and has a direct role in inhibiting insulin secretion.

### Pathways associated with changes in insulin secretion

#### Analysis of transcription factors involved in insulin secretion and islet proliferation

Based on the network analysis, C1ql3 is a potential regulator of islet function, with enriched ontology terms indicating a potential role in insulin secretion processes. To validate this finding, we determined whether C1ql3 regulates the expression of key transcription factors that are involved in islet function that may impact insulin secretion levels. The mRNA abundance of *Hnf4α, Hnf1*, *Egr1*, *Pdx1*, *MafA*, *NeuroD1*, *Lhx1*, and *Nkx6.1* was determined in INS1(832/13) β-cells expressing C1ql3 or GFP control. A significant reduction (p < 0.05) in the expression of *Pdx1, NeuroD1, MafA, and Nkx61* was observed in response to C1ql3 overexpression. However, the expression of other transcription factors, including *Egr1, Hnf4α, Hnf1, and LhX1*, remained unaltered in response to C1ql3 (data not shown), suggesting specificity in the C1ql3 effect.

Based on these gene expression results, we determined the effect of C1ql3 on the expression of key β-cell genes such as *Gck, Glut2, Kcnj6, and Kcnj11* that are regulated by *Pdx1, NeuroD1, MafA, or Nkx6.1*. Significant inhibition (p < 0.05) in the expression of *Gck, Glut2, and Kcnj6*, was observed in C1ql3 compared to control GFP expressing cells (Fig. 10j). These results suggest that C1ql3 inhibits the expression of transcription factors and their target genes that are key to the pancreatic islet function.

## Discussion

Co-expression network analysis along with data filtering approaches of gene expression data obtained from islets of lean and obese mice identified C1ql3 as a hub gene affecting islet function in obesity. Gene set enrichment analysis and hierarchical clustering of *C1ql3*-correlated transcripts putatively identified C1ql3 as a regulator of secretory processes in islets. Functional validation shows *C1ql3* is expressed in β-cells and it inhibits insulin secretion and the mRNA abundance of genes that are important in regulating pancreatic β-cell function. These findings, which are based on an unbiased analysis of lean and obese islet gene expression data and functional characterization identifies C1ql3 as a regulator of islet function in obesity. We posit that alterations in β-cell expression-coupled secretion of C1ql3 regulates insulin secretion in an autocrine and/or paracrine manner.

C1ql3 is a member of the complement-1q/TNF-related (CTRPs) family of secreted proteins^30–32^. These proteins contain 17 G-X-Y collagen repeats and a conserved C1q domain that are critical for their functional activity. Members of the CTRP family, such as adiponectin^2–4^, C1q/Tnf5^33^, and C1q/Tnf1^34^, are involved in regulating the whole body glucose homeostasis. Fasting or food restriction in mice decreases the expression of C1ql3 in the brain^35^. Conversely, diet-induced obesity (DIO), obesity caused by deficiency in leptin signaling, and agonists of PPARα (a fatty acid-activated transcription factor) increase the mRNA abundance of *C1ql3* ~3-fold in the adipose tissue^5^. Elevated C1ql3 levels were reported in the serum of B6-Ob mice as compared to B6-lean^5^, and epidemiology-based studies have associated human C1ql3 serum levels with an elevated risk of T2D^36,37^. These studies suggest nutritional and/or other changes related to obesity regulate the expression and secretion of C1ql3. Functionally, C1ql3 recombinant protein was reported to have an insulin-sensitizing effect^5,35^, suggesting a role in affecting glucose metabolism. However, the mechanism by which C1ql3 regulates glucose metabolism by modulating islet function remains uncharacterized.

Islet tissue constitutes less than 1% of the pancreas mass and is a very small tissue in the context of the whole body^38^. Therefore, islet-derived secreted proteins are poised to have an important regulatory function in an autocrine or paracrine manner to affect islet function within the pancreas. Our co-expression network-based analysis identified 18 enriched modules within lean and obese islet expression data. Within these modules, islet-derived candidate secreted proteins were identified and ranked based on their DE, IMC, and MM attributes as potential ‘hub genes’ (i.e., candidate regulators). 44 candidate secreted proteins were identified based on their high IMC and MM values with FC > 2 (DE, p < 0.05) in islets of lean and obese mice. Furthermore, 2 islet regulators were identified the lean condition and 12 in obesity by separately analyzing lean and obese islet expression data. By comparing regulators that were identified for lean, obese, and merged islet expression data, *Cck, C1ql3, Serpina7, Creld2, Svop, Smoc1, Tgfβ3, and Serpini1* were identified to affect islet function in obesity (Table 4). These islet-derived regulators are anticipated to mediate, at least in part, the effect of obesity on islet function by either alleviating or exacerbating the ability of islets to compensate for the stress of obesity. Understanding the role of these factors in islet biology will provide insights into obesity-induced T2D.

C1ql3 was identified as one of the top candidates that affect islet function in obesity. This ranking was based on high MM and IMC values of 0.92 and k_Total_ = 2455, respectively, within the islet expression data. The high connectivity of C1ql3 based on these attributes identified C1ql3 as a major “hub gene” affecting islet function in obesity. Ontology enrichment and hierarchical clustering identified C1ql3 to affect secretion processes from islets. Furthermore, C1ql3 is DE (>32-fold) with obesity, and inhibits insulin secretion and the expression of genes involved in β-cell function (Table 4 and Figs 8 and 10). These results suggest that islet-derived C1ql3 may contribute to reduced insulin secretion observed during impaired glucose tolerance, even before a clinical diagnosis of T2D.

The correlates of *C1ql3* in islets enriched for the GO terms associated with secretion and clustered by the expression levels to differentiate lean *vs*. obese mice. REVIGO analysis to determine the function of C1ql3 identified ‘regulation of secretion’ as a major supercluster in islets. This supercluster contained the highest frequency of all the ontology terms identified in islets for *C1ql3* correlates. The network of GO terms for this supercluster, determined by PANTHER, corresponded to functions such as cell migration, secretion, organelle localization, regulation of cellular catabolic process, regulation of hormone levels, negative regulation of molecular function, regulation of DNA metabolic process, ion homeostasis, and regulation of secretion. These results are indicative of the role of C1ql3 in regulating secretion from islets. This outcome is also supported by the cluster analysis of C1ql3 correlates identified in islets of lean and obese mice. Many of the *C1ql3* correlates that differentiated lean and obese animals were related to β-cell function and have been shown previously to be associated with obesity or T2D (Fig. 8 and Supplementary Files S12–S14). These genes included *Nucb2, Ins2, Cox2, Tcf7l2, Gcg, Ptprn, Sycn, Sytl4, Lin7c, Kcnq5, Unc2 (Munc18), Vdr, Syt13, Sdf4, Hap1, Slc2a2, Klk1, Dmbt1, Rnase1, Rbp4, Itm2c, Med31, Atp6, Peg3, Ppu, Cpa1, Reg1, Cpb1, Prss2, Ptchd2, and lapp*^39–43^. These outcomes support the role of C1ql3 as a regulator of β-cell function that modulates insulin secretion.

Other smaller superclusters identified for *C1ql3* in islets were related to the cellular response to endogenous stimulus, nuclear division, mitotic cell process, cell division, and protein phosphorylation. The number of GO terms representing each of these superclusters was lower as compared to the ‘regulation of secretion’ supercluster. The combined list of ontology terms for these superclusters was associated with organelle fission, nuclear division, negative regulation of programmed cell death, regulation of cell growth, regulation of cell projection organization, mitotic cell cycle, cell cycle phase transition, regulation of cell cycle, chromosome segregation, protein phosphorylation, and cell division (Fig. 8). Identification of these cellular functions suggests that the C1ql3 may function in affecting regulating islet mass. This inference is supported by the reduced expression of transcription factors that are key to regulating β-cell mass by C1ql3 (Fig. 10c).

In screening to determine the mechanism by which C1ql3 regulates islet function, we identified expression of key transcription factors—*Pdx1, NeuroD1, Nkx6.1*, and *MafA*—was decreased in response to the overexpression of C1ql3 in pancreatic β- INS1(832/13) cells. These transcription factors function to provide a link between pathways that regulate insulin secretion and pancreatic islet mass in differentiated β-cells and are known to regulate insulin secretion. Islets isolated from *Pdx1, MafA, NeuroD1, and Nkx6.1* deficient mice have reduced glucose-stimulated insulin secretion. Additionally, these factors also regulate β-cell growth during the metabolic stress of obesity, pregnancy, and age^44–46^. Islets from adult mice with 50% reduction in *Pdx1* expression were reported to have increased susceptibility to apoptosis, impairment in the ability to maintain β-cell mass with age, and reduction of IGF induced β-cell proliferation. Similarly, deletion of *Nkx6.1* in adult mice caused diabetes due to the reduction in insulin secretion and β-cell proliferation. The knockdown of *NeuroD1* in adult β-cells caused reversion to immature β-cells. Overexpression of C1ql3 caused a reduction in the expression of *Pdx1, NeuroD1, Nkx6.1, and MafA* in β-cells (Fig. 10). These results point to a mechanism by which C1ql3 can modulate both β-cell function and mass by regulating the expression and/or activity of key transcription factors. Delineating the mechanism of action of C1ql3 may identify C1ql3 as a master regulator of β-cell biology. Furthermore, islet cell transcriptional regulation by C1ql3 may provide insights into the mechanism underlying long-term nutritional adaptation of β-cells during obesity and may provide insight into why only a fraction of obese individuals develop T2D. The mechanism by which C1ql3 regulates islet insulin secretion will be the subject of future studies.

The ability of β-cells to adapt to the stress of obesity is critical for the maintenance of whole-body glucose homeostasis and prevention of T2D. Typically, during obesity, fasting glucose levels are increased, which prompts β-cells to increase their mass and insulin secretion to maintain euglycemia. The inability of β-cells to compensate for obesity stress leads to a reduction in functional in β-cell mass, and, ultimately, T2D^13,47^. Therefore, identification and characterization of regulatory factors that mediate the reduction in functional β-cell mass during obesity will provide critical insights into the mechanisms underlying progression to T2D. C1ql3 is expressed in β-cells, its expression is elevated in islets obtained from mouse models of obesity (HFD-induction, Ob/Ob, and Db/Db), and it inhibits insulin secretion from mouse islets and INS1(832/13) β-cells. These results suggest C1ql3 functions in an autocrine or paracrine manner to negatively regulate insulin secretion from β-cells. Correlation and ontology-based analyses identified C1ql3 as a hub gene with high IMC to affect islet function in obesity by modulating secretion, nuclear division, mitotic cell process, and cell division. These outcomes identified C1ql3 as an important metabolic regulator of islet function that may be involved in modulating both function and mass of β-cells. An important next step will be to determine the role of C1ql3 in balancing β-cell mass and insulin secretion during lean and obese states. These studies are currently being investigated.

In sum, an unbiased data filtering approach identified novel and previously known regulators of islet function in the lean and obese states based on their change in response to obesity (differential expression), magnitude of change (fold change), statistical significance of networks identified by WGCNA (association of modules with obesity), and ranking as regulator/hub genes (module membership). Within this list of candidate gene regulators, genes were additionally filtered based on their overall connectedness to the networks (k_Total_ rank) when using the combined data or lean and obese data separately. This final list of regulators identified known and novel regulators of metabolic processes related to islet function in obesity that would impact the susceptibility to T2D. Importantly, the identification of novel regulators of islet function demonstrates that the gene expression data can be a valuable resource to effectively screen for the novel secreted protein regulators. Our approach could also be used to identify regulators such miRNA, metabolites, natural or synthetic compounds that affect islet function. Identifying such factors that function to contribute towards the disease risk during obesity will be useful in translational medicine as they will improve the risk assessment of the disease state, and their functional characterization will lead to the identification of therapeutic drug targets in obesity.

## Materials and Methods

### Statistical analysis of microarray data

Microarray data from 10-week old lean and obese mice (GEO accession: 10785) was used for analysis in this study^21^. All normalized data (i.e., intensity2 or mlratio) are publicly available for download at the http://diabetes.wisc.edu/search.php website. All results from the differential expression analysis were derived from Keller *et al*. as identified on the website above^21^. Differentially expressed (DE) genes were selected based on q-values (q < 0.05) (Supplementary File S3). Expression levels used for correlation analysis were derived from intensity2 data that was log normalized.

### Weighted gene co-expression network analysis

The weighted gene co-expression network analysis (WGCNA) package in R version 1.63 was used to identify the group of modules representing co-expressed networks of genes in the expression data that was obtained from islets of the lean and obese mice^23^. The determination of the co-expression network was based on first generating a similarity correlation matrix, which was calculated for all the genes in the data by performing a pairwise Pearson’s correlation [cor(i, j)]. The similarity matrix was then transformed to an adjacency matrix (AM) (A = [aij] where aij = Sijβ), which is comprised of an unsigned weighted gene co-expression network by using the power β = 6 (soft thresholding) (Supplementary Fig. 2). The soft threshold function in WGCNA was used to determine the weightiness of the edges connecting the sets of the genes to retrieve stronger results. Soft thresholding establishes the robustness of correlation of genes within a module at a particular power threshold to demonstrate correspondence to scale-free network topology. We identified a soft threshold of β = 6, which was chosen based on the criteria of approximating the scale-free topology of the network^48^, where R^2^ = 0.78. Increasing the power *via* this method to or above the soft-threshold reduces/removes the noise in the correlation network (adjacency matrix), and no sample outliers were identified in the data. To generate a gene dendrogram, the topological overlap matrix (TOM) and *diss*TOM = (1-TOM) were calculated. Hierarchical clustering based on a Tree cutHeight of 0.2 (based on the *diss*TOM = (1-TOM)) was used to identify gene clusters within the data. Next, the module eigengene (ME) value was calculated based on the first principal component for the entire expression profile for each module. This analysis was computed using the *blockwiseModules* function in WGCNA with deepsplit = 3 and minimum module size = 100. Each module was assigned an ID corresponding to a unique color name. The MEs were used in performing module–trait relationship (correlation) analysis and to determine the intramodular connectivity (IMC) and module membership (MM) statistics. The parameters described above were used for generating modules and their MEs, module-trait relationships, MM, and IMC in the lean and obese islet expression data, when analyzed as separately or as merged.

### Module-trait relationships

To determine whether the modules in islets were significantly associated with the lean and obese traits, a binary matrix was generated describing the association of the samples with their respective traits (case (obese) = 1, control (lean) = 0). This matrix was then used as an input file for phenotype traits. The MEs calculated above were used to calculate the p-value (asymptotic) and correlation (calculated using Pearson correlation) value for lean and obese traits. Modules correlated with traits in lean and/or obese islets were filtered based on p < 0.05.

### Intramodular connectivity (IMC) and module membership (MM)

A connectivity measure (k_Total_) was calculated for each candidate regulatory gene by determining the strength of the connection of a gene with the other genes across the enriched modules. Moreover, the k_Within_ was calculated for each gene within their respective module. The genes in the top 25 percentile based on the k_Total_ ranking were used to make a reference list and hierarchical clustering and GSEA to identify regulators of islet function. Another measure of connectivity for the gene is the (fuzzy) module membership (MM). The MM was based on the Pearson correlation between the expression profile of each gene and each ME. A larger value indicates greater similarity between the gene and its respective ME. Genes with high MM (MM > 0.80) were considered as hub genes, as they are highly correlated with the other genes in their module^49^.

### Identification of differentially expressed genes

An R-script was used to identify secreted proteins that were differentially expressed (DE) (q < 0.05) with higher than average log10 expression value across all the lean and obese samples.

### Gene set ontology analysis (GSEA)

Highly correlated transcripts (p < 0.05) with correlation value (r>|0.5|) were used in the GSEA. Significantly correlated transcripts to a specific candidate regulator were tested for Gene Ontology (GO) enrichment^50,51^. GO enrichment analysis was conducted with the GO and KEGG packages of R as well as the Panther ontology tool (http://pantherdb.org/). Significant GO terms (Bonferroni adjusted p < 0.05) containing less than 200 transcripts were selected to generate superclusters based on the grouping of the common terms by using the REVIGO software^28^.

### Expression constructs

The untagged C1ql3 mammalian expression plasmid was generated by cloning C1ql3 cDNA (ATCC) into an MMLV-based lentiviral vector (LVV, 3565) (a gift from Dr. Bill Sugden, University of Wisconsin, Madison, WI, USA).

### Cell culture and transient transfection

INS1(832/13) pancreatic β-cells (a gift from Dr. Christopher Newgard, Duke University, NC, USA) were cultured in supplemental RPMI 1640 media containing 10% heat-inactivated fetal bovine serum, 2 mM L-glutamine, 1 mM sodium pyruvate, 10 mM HEPES, and 100 U/ml of antimycotic-antibiotic along with 50 µM β-mercaptoethanol. Approximately 8 million cells were plated in each 60 mm tissue culture-treated dish. Next day, cells at 75–80% confluency were transfected with 5 µg of the C1ql3 or GFP expressing plasmid by Lipofectamine 2000 (Invitrogen) at a ratio of 1:1 in OPTIMEM cell culture medium. After 40 h, cells were harvested for RNA analysis. For insulin secretion, ~500,000 and 100,000 cells were plated in each well of a 24- and 96-well-plate, respectively. Next day, cells were transfected with 20 µM siScramble (Control) or siC1ql3 using 2 µl Lipofectamine 2000 in OPTIMEM cell culture medium. After 36 h, cells were harvested for RNA or static insulin secretion analysis.

Mouse islets were isolated from 10–12-week-old C57BL/6J male or female mice using a collagenase digestion method^52^. Isolated islets were cultured overnight in supplemented RPMI 1640 containing 8 mM glucose. After 16 h, size-matched islets were hand-picked for static insulin secretion assays. For adenoviral-mediated transduction, ~200 islets were infected with either 200 MOI of C1ql3 or GFP expressing adenovirus in supplemented RPMI 1640 containing 8 mM glucose. After 40 h, size-matched islets were hand-picked for static insulin secretion assay or RNA analysis.

### Insulin secretion in INS1(832/13) and mouse islets

Static insulin secretion in INS1(832/13) pancreatic β-cells was performed in Krebs-Ringer Bicarbonate based buffer (KRB: 118.41 mM NaCl, 4.69 mM KCl, 1.18 mM MgSO_4_, 1.18 mM KH_2_PO_4_, 25 mM NaHCO_3_, 20 mM HEPES, 2.52 mM CaCl_2_, pH 7.4, and 0.2% BSA) containing  3 mM glucose as described previously^53^. Briefly, INS1(832/13) cells were pre-incubated in 100 µl of KRB-HEPES-based buffer containing  3 mM glucose for 2 h. Next, the pre-incubation buffer was aspirated and replaced with 100 µl of KRB-HEPES based incubation buffer containing  3 mM or 15 mM glucose. Insulin secreted in the incubation media in response to treatment was quantified by using an in-house insulin ELISA and was expressed as a percent of the total insulin content (secreted plus cellular insulin).

Six size-matched mouse islets were handpicked for secretion assay into each well of a 96-well-plate. The procedure was performed as described previously^52^. Briefly, islets were preincubated in 100 µl of RPMI 1640 containing 3 mM glucose for 45 min. Next, the pre-incubation media was removed and replaced with incubation media containing insulin secretagogues for another 45 min. Culture media and islets were processed to determine insulin secreted and cellular insulin content, respectively. For insulin content, islets were harvested using acid-ethanol. The protein abundance of insulin in the media and islets was estimated using an in-house ELISA. The insulin secreted was normalized to the total insulin (secreted plus content) and the data was represented as a percent of total.

### Isolation and quantitation of total RNA

Total RNA was harvested from mouse tissues and INS1(832/13) cells by using a QIAGEN RNeasy Plus Kit. Following extraction, RNA was used for cDNA synthesis (Applied Biosystems). The mRNA abundance for the gene of interest was determined by quantitative PCR using Fast Start SYBR Green (Roche). The data was quantified by the comparative ΔCT method and expressed relative to β-actin mRNA. Data were expressed as means ± standard error of means. Statistical comparisons were made using Student’s *t-*test at *p* < 0.05.

### Isolation and quantitation of protein

Cells were lysed in 20 mM Tris-HCl (pH 7.5), 150 mM NaCl, 1 mM Na_2_EDTA, 1 mM EGTA, 1% Triton, 2.5 mM sodium pyrophosphate, 1 mM β-glycerophosphate, 1 mM Na_3_VO_4_, 1 mM PMSF, and protease inhibitor cocktail tablet (Roche). The extraction of soluble proteins, quantitation, and western blot analysis was performed as described previously^54^.

### Immunofluorescence

Pancreatic tissue from C56BL/6J mice was fixed overnight in paraformaldehyde, paraffin-embedded, and sectioned with a microtome at 6-micron thickness. Sections were placed onto a microscope slide to prepare for staining. After blocking with 10% normal donkey serum, the slides were incubated overnight with primary antibodies in dilution buffer of 2% BSA in PBS at 4 °C. Anti-C1ql3 antibody (rabbit, 1:500) (Invitrogen, #PA5-75736), anti-insulin antibody (guinea pig, 1:500) (DAKO, #A0564), anti-glucagon antibody (mouse, 1:500) (Sigma, #G2654), and anti-somatostatin antibody (goat, 1:500) (Santa Cruz Biotechnology, #sc-7819) were used for staining. The next day, a secondary antibody was applied to the section for 2 h at room temperature. Anti-Cy5 (rabbit, 1:500) (Jackson ImmunoResearch, #711-175-152), anti-Cy3 (guinea pig 1:500) (Jackson ImmunoResearch, #706-165-148), anti-Cy3 (mouse, 1:500) (Jackson ImmunoResearch Catalog 715-165-161), and anti-Cy3 (goat, 1:500) (Jackson ImmunoResearch, #705-165-003) secondary antibodies were used. Finally, a coverslip was mounted on top of the section using mounting buffer with DAPI and sealed with nail polish. The slides were visualized using an epi-fluorescent microscope at 1376 × 1038-pixel resolution at a total magnification of 25.2. Viewing channels include Cy5 to view C1ql3, Cy3 to view insulin, glucagon, and somatostatin, and DAPI to view the nucleus.

### Ethics approval and consent to participate

Not applicable. The data used in this manuscript was derived from Keller *et al*., (2008)^21^.

## Supplementary information

Supplementary Data

Dataset 1

Dataset 2

Dataset 3

Dataset 4

Dataset 5

Dataset 6

Dataset 7

Dataset 8

Dataset 9

Dataset 10

Dataset 11

Dataset 12

Dataset 13

Dataset 14

Dataset 15

## Data Availability

Microarray data for this study is available from GEO accession #GSE10785 (Keller *et al*.)^21^. All normalized data (i.e., intensity2, mlratio) are publicly available for download at the http://diabetes.wisc.edu/search.php website. All results from the differential expression analysis are derived from Keller *et al*.^21^.
