# Supplementary material for: A gene expression network analysis of the pancreatic islets from lean and obese mice identifies complement 1q like-3 secreted protein as a regulator of β-cell function
Source: Sci Rep. 2019 Jul 12;9:10119. doi: 10.1038/s41598-019-46219-3 (PMC6626003; doi:10.1038/s41598-019-46219-3)
Supplement: Supplementary file 1 — Supplementary Data [file 41598_2019_46219_MOESM1_ESM.pdf]

A gene expression network analysis of the pancreatic islets from lean and obese mice identifies complement 1q like-3 secreted protein as a regulator of β-cell function

James E. Koltes[1#], Itika Arora[2#], Rajesh Gupta[2#], Dan C. Nguyen[2], Michael Schaid[3,7], Jeong-a Kim[2], Michelle E. Kimple[3,4,5,6,7], and Sushant Bhatnagar[2*]

[1]Department of Animal Science, Iowa State University, Ames, IA 50011, USA

[2]Division of Endocrinology, Diabetes, and Metabolism, Department of Medicine and Comprehensive Diabetes Center, University of Alabama, Birmingham, AL 35294, USA

[3]Interdisciplinary Graduate Program in Nutritional Sciences, University of Wisconsin-Madison College of Agriculture and Life Sciences, Madison, WI 53706, USA

[4]Divison of Endocrinology, Diabetes, and Metabolism, Department of Medicine, University of Wisconsin-Madison School of Medicine and Public Health, Madison, WI 53705, USA

[5]Department of Cell and Regenerative Biology, University of Wisconsin-Madison School of Medicine and Public Health, Madison, WI 53705, USA

[6]Department of Academic Affairs, University of Wisconsin-Madison School of Medicine and Public Health, Madison, WI 53705, USA

[7]Research Service, William S Middleton Memorial VA Hospital, Madison, WI 53705, USA

Supplementary Fig S1

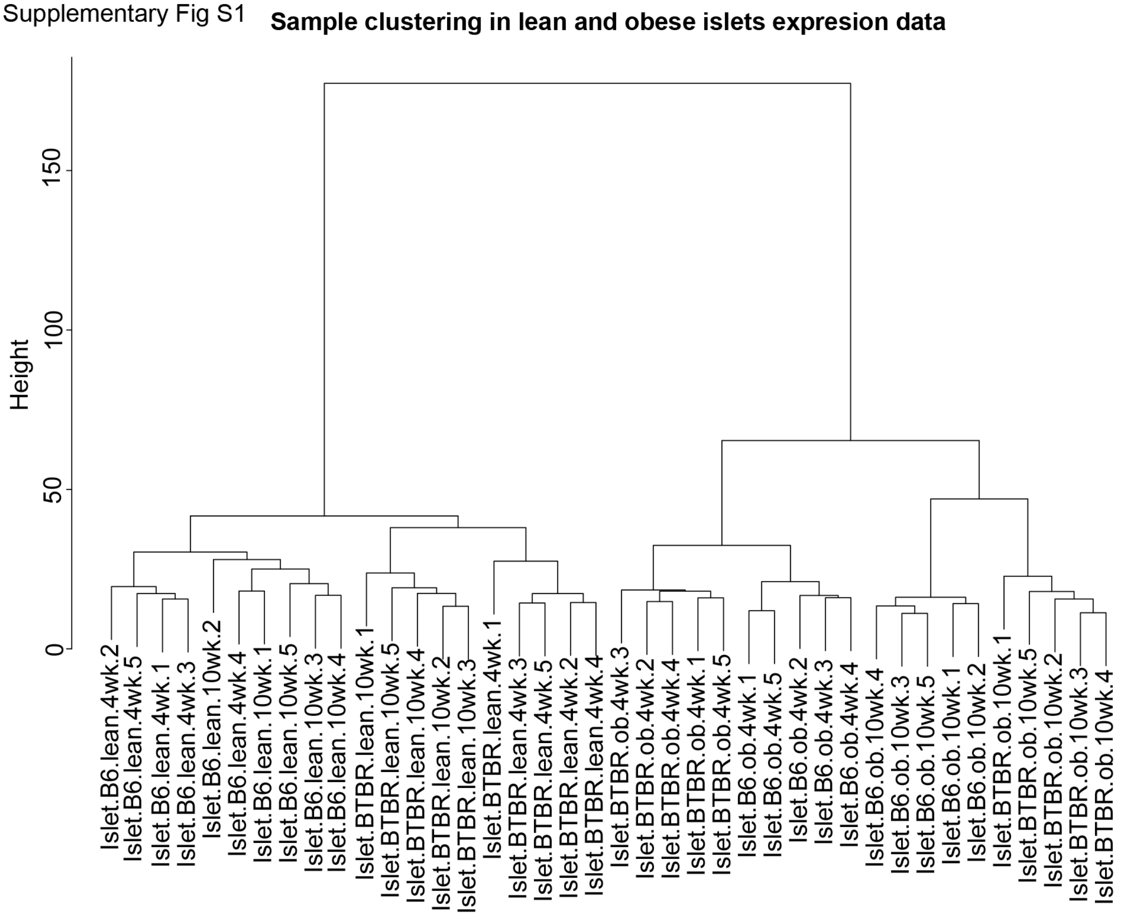

Supplementary Figure S1. Sample clustering of lean and obese islet gene expression data.

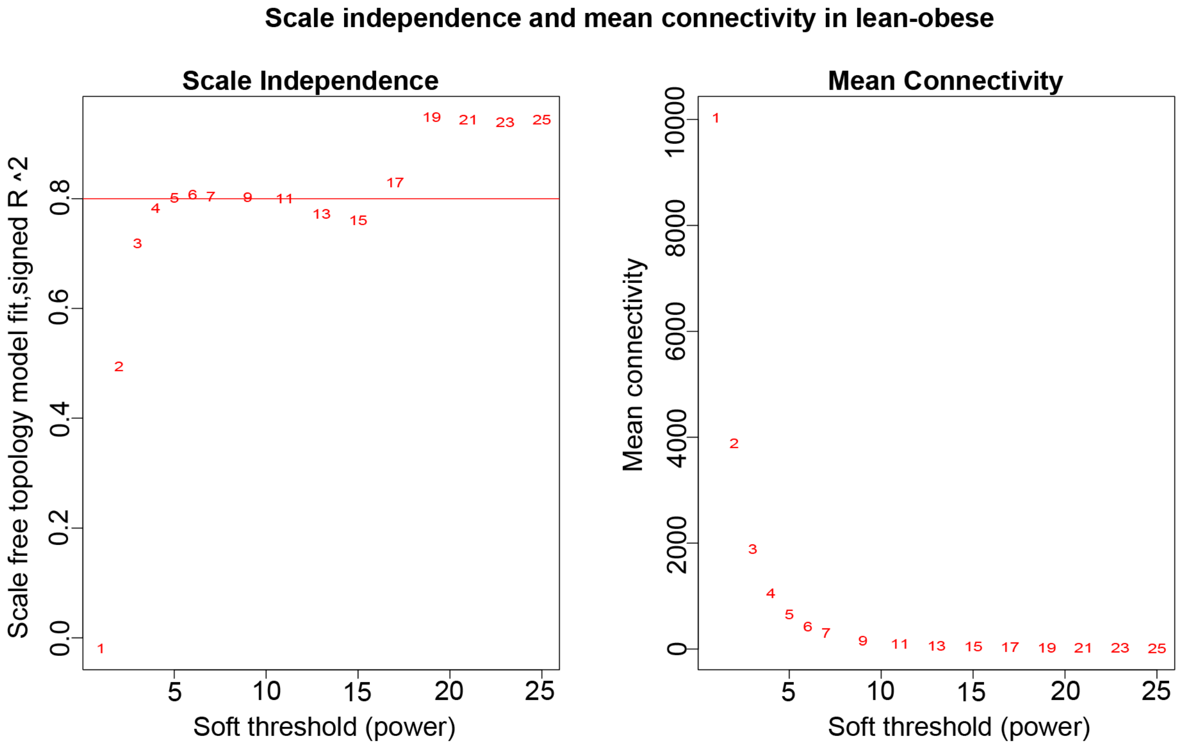

Supplementary Figure S2. The soft threshold selection parameter for lean and obese islet gene expression data. The numbers in the plot indicate soft thresholding powers. The scale-free topology was determined as the point where the soft threshold power intersects at 0.8 (cut height=0.2) on the y-axis.

a) Pathway prediction for somatostatin in slets

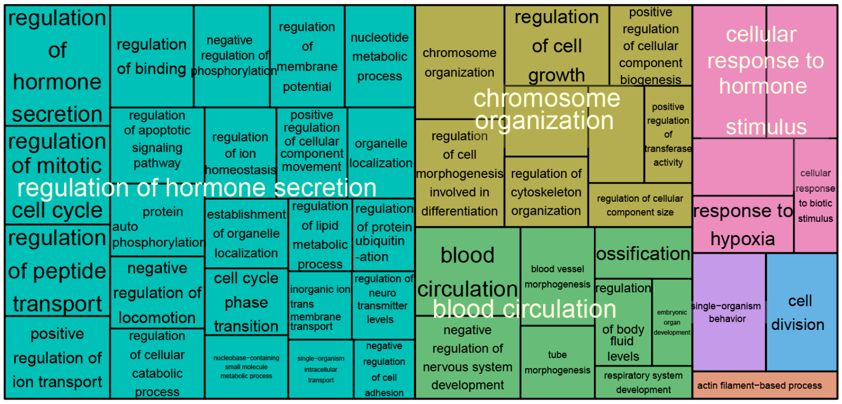

b) Pathway prediction for Tff2 in islets

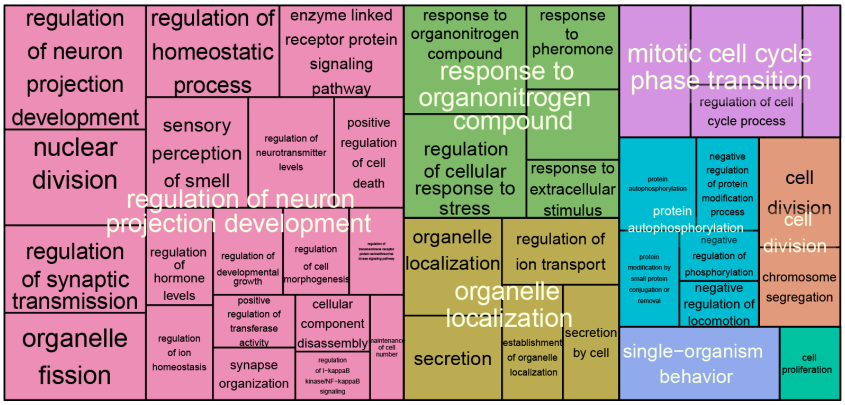

Supplementary Figure S3. Pathway prediction using REVIGO Gene Ontology enrichment analysis. A Tree plot generated by REVIGO software showing enriched functions for (a) somatostatin and (b) TFF2 in islets.

a)

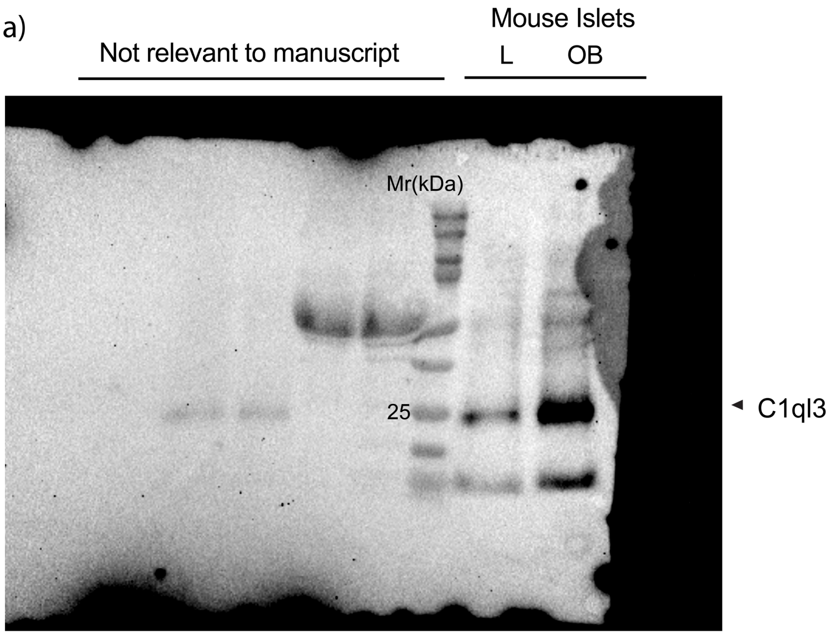

b)

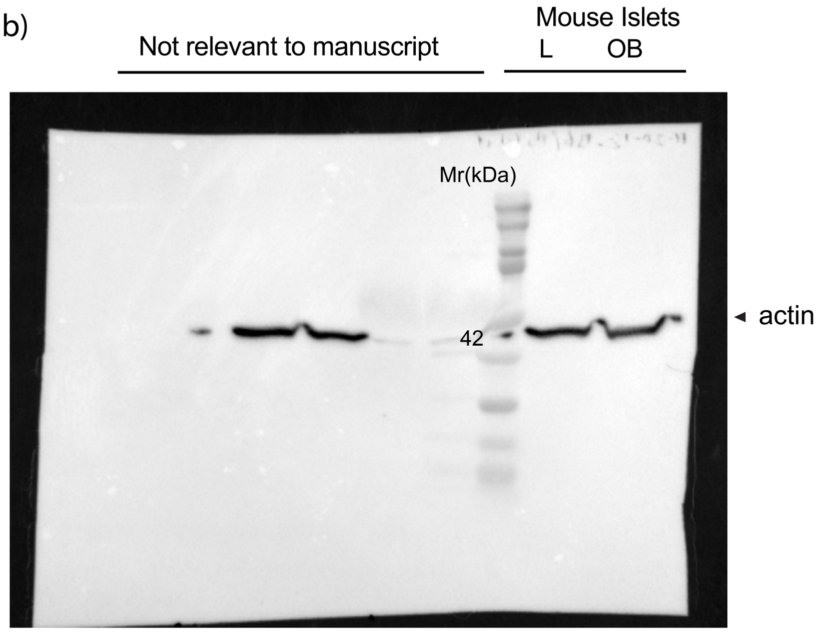

Supplementary Figure S4. Full length gel image of the blot shown in Figure 10e.

a) b)

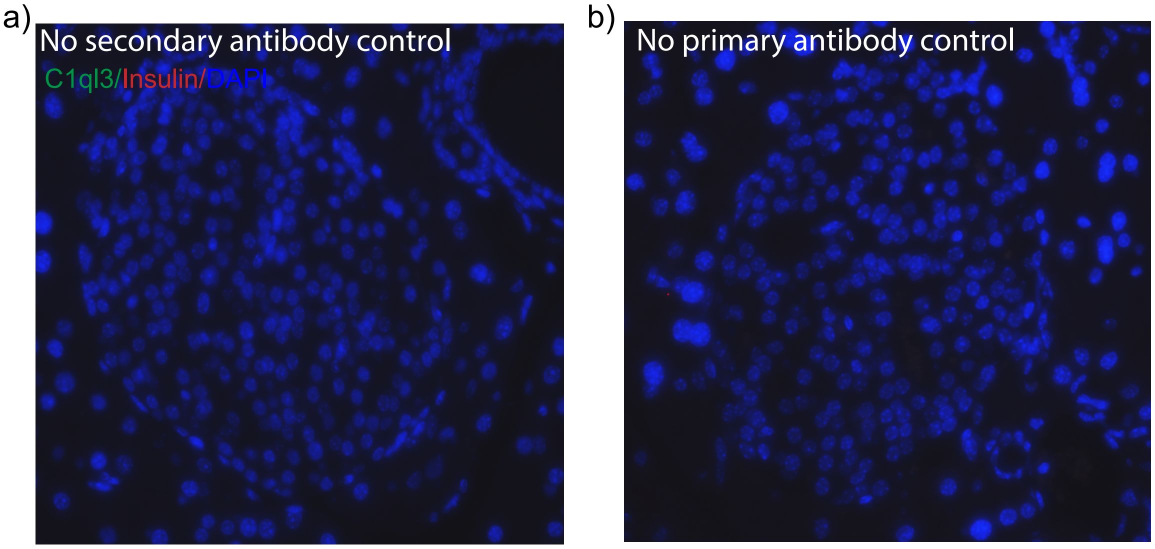

Supplementary Figure S5. a) Immunofluorescence of pancreatic section using primary antibodies for C1ql3, insulin, and DAPI in the absence of secondary antibodies. b) Immunofluorescence of pancreatic section with secondary antibodies in the absence of primary antibodies for insulin and C1ql3 proteins.

Supplementary Table S1: Intramodular connectivity (IMC) and modular membership (MM) values for genes that enriched in modules that were identified by analyzing merged lean and obese islets expression data. The reference list was also generated for genes that had high IMC with MM (>0.8).

Supplementary Table S2. A list of annotated secreted proteins.

Supplementary Table S3. A list of differentially expressed (DE) genes with obesity (q<0.05) in islets.

Supplementary Table S4. A list of differentially expressed secreted protein transcripts with q<0.05 with calculated fold change of the mRNA expression with obesity.

Supplementary Table S5. A list of differentially expressed secreted transcripts with q<0.05, IMC, and MM (>0.8) calculated by using merged lean and obese islets expression data.

Supplementary Table S6. Modules identified by using lean islet expression data.

Supplementary Table S7. Modules identified by using obese islet expression data.

Supplementary Table S8. Intramodular connectivity (IMC) and modular membership (MM) values for genes that enriched in modules that were identified by analyzing lean islet expression data. The reference list was also generated for genes that had high IMC with MM (>0.8).

Supplementary Table S9. Intramodular connectivity (IMC) and modular membership (MM) values for genes that enriched in modules that were identified by analyzing obese

islet expression data. The reference list was also generated for genes that had high IMC with MM (>0.8).

Supplementary Table S10. A list of differentially expressed secreted transcripts with q<0.05 and MM (>0.8) calculated using islet expression data from lean mice.

Supplementary Table S11. A list of differentially expressed secreted transcripts with q<0.05, IMC, and MM (>0.8) calculated using islet expression data from obese mice.

Supplementary Table S12. A list of GO terms identified from C1ql3 islet correlates.

Supplementary Table S13. A list of all ontology terms used in the REVIGO analysis.

Supplementary Table S14. Superclusters identified for C1ql3 in islets using REVIGO software.

Supplementary Table S15. Superclusters identified for Tff2 and Sst in islets using REVIGO software.

Primers

Target gene	Primer sequence
rat INS1 and INS2	F: GTCATTGTTCCAACATGGCCCTGT
	R: TGCAGTAGTTCTCCAGTTGGTA
rat GK	F: GGAAGACCTGAAGAAGGTGATG
	R: TCCCAGGTCTAAGGAGAGAAAG
rat Glut2	F: FTCTTCACGGCTGTCTCTGTG
	R: GAAGATGGCCGTCATGCTCA
rat PDX1	F: GCAGAACCGGAGGAGAATAAG
	R: GGCCGGGAGATGTATTTGTTA

ratMAFA	F: GGTCATCCGACTGAAACAGAA
	R: CTTCTCGCTCTCCAGAATGTG
ratNeuroD1	F: GAACACGAGGCAGACAAGAA
	R: TCATCTTCATCCTCCTCCTCTC
rLin7c	F: GCCCTGAAGTGAGAGCTAATG
	R: TATTGAATCCAAGGCCCTCTTC
ratNKX6.1	F: TGGACAAAGATGGGAAGAGAAA
	R: TCTCTGGTCCTGCCAAGTA
Kcnj6	F: CTCGTGTCACCGCTGATTATTA
	R: CCACAATCTCCAGTTCCTCTTT

List of abbreviations

ATP6 = ATP synthase F0 subunit 6

β-cell = pancreatic β- cells

C1ql3/ CTRP13 = Complement 1q-like 3

Cckar = cholecystokinin A receptor

Cfd = adipsin

COX2 = cytochrome c oxidase subunit II

Cpa1 = carboxypeptidase A1, pancreatic

Cpb1 = carboxypeptidase B1, pancreatic

CTRPs = complement 1q/TNF-related family

DAG = diaclglycerol

dC_t = delta cycle threshold

DE = differentially expressed

Dmbt1 = deleted in malignant brain tumors 1

DMEM = Dulbecco's modified Eagle's medium

ELISA = enzyme-linked immunosorbent assay

Epac2 = exchange protein directly activated by cAMP 2

GAIN = G-protein-coupled receptor (GPCR) autoproteolysis-inducing domain

Gcg = glucagon

Gcgr = glucagon receptor

GFP = green fluorescent protein

GLP-1 = glucagon like peptide-1

GO = gene ontology

GWAS = genome-wide association study,

Hap1 = huntingtin-associated protein 1

Ins2 = insulin2

Itm2c = Integral Membrane Protein 2C

K_{ATP} = potassium adenosine triphosphate

Kcnq5 = Potassium Voltage-Gated Channel Subfamily Q Member 5

Klk1 = Kallikrein-1

KRB = Krebs Ringer Buffer

Iapp = Islet amyloid polypeptide

Lin7c = Lin-7 Homolog C, Crumbs Cell Polarity Complex Component

MAG = Monoacylglycerol

mRNA = messenger ribonucleic acid

Med31 = Mediator Complex Subunit 31

MMP12 = Metallopeptidase 12

Munc18 = Mammalian homologue of UNC-18

Nrxn1 = Neurexin 1

Nucb2 = nesfatin

Pon1 = paraoxonase 1

PDZd11 = Plasma Membrane Calcium ATPase-Interacting Single-PDZ Protein

PEG3 = Paternally-Expressed Gene 3 Protein

PKA = protein kinase A,

Ppu = Protein phosphatase U

Prss2 = Protease, Serine 2

Ptchd2 = Patched Domain Containing 2

Ptprn = Protein Tyrosine Phosphatase

Rab11a = Member RAS Oncogene Family

Rbp4 = Retinol Binding Protein 4

Reg1 = Regenerating islet-derived 1

RPMI 1640 = Roswell Park Memorial Institute 1640 medium

Rnase1= Ribonuclease A Family Member 1, Pancreatic

Sdf4 = Stromal Cell Derived Factor 4

Slc2a2 (GLUT2) = Solute Carrier Family 2 Member 2

Snap9 = Soluble NSF attachment protein

SNPs = single nucleotide polymorphisms,

Stxbp2 = Syntaxin Binding Protein 2

Stxbp5 = Syntaxin Binding Protein 5

Sycn = Syncoilin

Sytl4 = Synaptotagmin Like 4

Syt9 =Synaptotagmin-9

Syt11= Synaptotagmin-11

Syt13 = Synaptotagmin-13

T2D = type 2 diabetes

Tcf7l2 = Transcription factor-7–like 2

TOM = Topological Overlap Matrix

TRS = N-terminal thrombospondin type 1 repeat

Vamp4 = Vesicle Associated Membrane Protein 4

Vdr = vitamin D receptor

WGCNA = Weighted Gene CO-expression Network Analysis
